# Early Marriage among  young  girls in Eastern Ethiopia: trend during 2008-2018

**DOI:** 10.12688/f1000research.55551.1

**Published:** 2021-08-16

**Authors:** Dureti Abdurahman, Nega Assefa, Yemane Berhane

**Affiliations:** 1 Department of Public Health, College of Health and Medical Sciences, Haramaya University, Harar, Ethiopia; 2 Department of Epidemiology and Biostatistics, Addis Continental Institute of Public Health, Addis Ababa, Ethiopia

**Keywords:** Key words:   adolescent girls, incidence, early marriage, Ethiopia

## Abstract

Early marriage practices undermine girls’ autonomy and seriously affect their physical and mental wellbeing. Monitoring the trends and understanding the drivers is essential in intervening against early marriage. However, many studies on early marriage in Ethiopia are cross-sectional, focusing only on the magnitude at a single point in time. Hence, we extracted data of girls of 10-17 years from Kersa Health and Demographic Surveillance System (Kersa HDSS) database for the period of 2008–2018 in order to examine the trends of early marriage. In this data note we provide the details of a research database of 24,452 girls in the age group of 10-17 years. The extracted data include date of marriage and the girls’ socio-demographic variables. Other variables considered to be potentially associated with timing of marriage were also extracted. The purpose of this publication is to describe the dataset for external researchers who may be interested in making use of it as a secondary use of their routinely collected data. This dataset is available at https://doi.org/10.6084/m9.figshare.15034812.

## Introduction

Early marriage is any marriage where at least one of the parties is under 18 years of age ( OHCHR, 2019). It undermine girls’ autonomy and seriously affects their physical and mental wellbeing ( Nour, 2006; Walker, Mukisa, Hashim, & Ismail, 2013). Ethiopia ranks 15th in the prevalence of early marriage and 5th in the total number of early marriages globally. Nearly 40% of girls in Ethiopia are married before they turn 18 years and approximately 14% are married before their 15th birthday ( UNICEF, 2018). Monitoring the trend and understanding the drivers is essential in intervening against early marriage. However, evidence on the effectiveness of interventions from longitudinal community-based studies is scarce. Hence, we extracted data of girls of 10–17 years from the Kersa Health and Demographic Surveillance System (Kersa HDSS) database for the period of 2008–2018 in order to examine the trends of early marriage.

## Methods

This data note used data from Kersa Health and Demographic Surveillance System (Kersa HDSS). The Kersa HDSS is located in the eastern Hararghe Zone of the Oromia regional state in Ethiopia. The initial baseline household and population census’ were conducted in 2007, and the database is updated every six months with registration of demographic (birth, death and migration) and health (reproductive and morbidity) events ( Assefa et al., 2016). The data are collected by trained interviewers who are mainly residents in the study Kebele (the smallest administrative unit in Ethiopia). In each round of data collection, the household head or any adult member of the household is interviewed using structured forms that are prepared to capture a specific demographic or health event.

### Source of data

Data was extracted from Kersa HDSS database for the period of January 01, 2008 to December 31, 2018 for girls in the age group of 10 to17 years. The extracted data includes date of marriage and girl’s socio-demographic variables. Other variables considered to be potentially associated with the timing of marriage were also extracted. Microsoft Excel 2010 (Microsoft Excel, RRID:SCR_016137) was used to process the data (an open access alternative to Excel 2010 is Google Sheets).

### Ethical approval

Kersa HDSS has ethical approval from the Institutional Health Research Ethics Review Committee (IHRERC) of Haramaya University at the initiation of the surveillance system and renewed every five years. The approval written informed consent for KHDSS head office for the sharing of girls’ data was obtained from the IHRERC of Haramaya University, Ethiopia with approval number (IHRERC/177/2018). The accessed data were used for this research only.

## Data availability

Datasets are available publicly via:

Figshare: Early Marriage among young girls in Eastern Ethiopia: trend during 2008-2018.

https://doi.org/10.6084/m9.figshare.15034812 ( Abdurahman et al. 2021).

The project contains the following underlying data.

• Early marriage data.xlsx. (contains data in excel spreadsheet of ten years of marriage data and girl’s socio-demographic variable)

Data are available under the terms of the Creative Commons Attribution 4.0 International license (CC-BY 4.0).

## Author contributions

All authors contributed equally from conception, design, data extraction, and statistical analysis to interpretation of data. They also took part in the drafting of the manuscript and final approval for submission.
