# Supplementary material for: Early Marriage among  young  girls in Eastern Ethiopia: trend during 2008-2018
Source: F1000Res. 2022 May 12;10:807. Originally published 2021 Aug 16. [Version 2] doi: 10.12688/f1000research.55551.2 (PMC10345594; doi:10.12688/f1000research.55551.2)
Supplement: Peer review history

# Peer review: Reviewer response for version 2

**DOI:** 10.5256/f1000research.133886.r181996

**Published:** 2023-07-13

**Authors:** Sarni Berliana

**Affiliations:** 1 Sekolah Tinggi Ilmu Statistik, Jakarta, Indonesia

The Kersa HDSS dataset for 2008-2018 includes more than 80,000 observations. The available variables include ethnicity, religion (Muslim, other), occupation (student, unemployed, other), place of residence (urban/rural), education level, age at survey (10-14, 15-17), and trend at marriage (10-14, 15-17). The authors admitted that the data lack of some important variables related to early marriage. However, the available data has relevant information that can be used for research on early marriage.

Are sufficient details of methods and materials provided to allow replication by others?

Yes

Is the rationale for creating the dataset(s) clearly described?

Yes

Are the datasets clearly presented in a useable and accessible format?

Yes

Are the protocols appropriate and is the work technically sound?

Yes

Reviewer Expertise:

Statistics, Population Studies

I confirm that I have read this submission and believe that I have an appropriate level of expertise to confirm that it is of an acceptable scientific standard.

# Peer review: Reviewer response for version 2

**DOI:** 10.5256/f1000research.133886.r137597

**Published:** 2022-06-21

**Authors:** İlknur Yüksel-Kaptanoğlu

**Affiliations:** 1 Institute of Population Studies, Social Research Methodology Department, Hacettepe University, Ankara, Turkey

Thank you for the revisions.

Are sufficient details of methods and materials provided to allow replication by others?

No

Is the rationale for creating the dataset(s) clearly described?

Yes

Are the datasets clearly presented in a useable and accessible format?

Yes

Are the protocols appropriate and is the work technically sound?

Yes

Reviewer Expertise:

Domestic violence against women, child, early and forced marriages, qualitative research methods, gender equality

I confirm that I have read this submission and believe that I have an appropriate level of expertise to confirm that it is of an acceptable scientific standard.

# Peer review: Reviewer response for version 1

**DOI:** 10.5256/f1000research.59137.r91981

**Published:** 2021-09-08

**Authors:** İlknur Yüksel-Kaptanoğlu

**Affiliations:** 1 Institute of Population Studies, Social Research Methodology Department, Hacettepe University, Ankara, Turkey

This data note describes the data set of Kersa Health and Surveillance System (Kersa HDSS) for the use of secondary analysis. It is appropriate for the publication in F1000 research. However, some parts of this data note needs information to explain the data set in a more detailed way before indexing.

 My minor concerns are the following:

- Introduction section should be elaborated by explaining the benefits of a data set for researchers who study child marriages. The definitions of early marriage, and the choice of using early marriage instead of child marriage should be explained. Since, in many data sets it is measured by the age of marriage before 18 or 15. The reference time should be included about the prevalence of child marriage of Ethiopia.
- In the material and method section (it must be named as), more explanation is needed about the system of KERSA HDHSS. The aim of the system in general, sample size, how data is collected, what are the other specific demographic events, other variables and what are the limitations of the data set some of the questions that should be mentioned.
- Some minor grammar mistakes should be reviewed.

Are sufficient details of methods and materials provided to allow replication by others?

No

Is the rationale for creating the dataset(s) clearly described?

Yes

Are the datasets clearly presented in a useable and accessible format?

Yes

Are the protocols appropriate and is the work technically sound?

Yes

Reviewer Expertise:

Domestic violence against women, child, early and forced marriages, qualitative research methods, gender equality

I confirm that I have read this submission and believe that I have an appropriate level of expertise to confirm that it is of an acceptable scientific standard, however I have significant reservations, as outlined above.

# Response

**Published:** 2021-12-01

**Authors:** Dureti Abdurahman

**Affiliations:** Haramaya University, Ethiopia

**Dear reviewer**

 We greatly appreciate the time and effort put forth to improve our paper. We benefited a lot from their insightful comments and suggestions. Based on the suggestions, we have provided a response to each comment as follows, and we will incorporate it on the main document.

***General comment***

*This data note describes the data set of Kersa Health and Surveillance System (Kersa HDSS) for the use of secondary analysis. It is appropriate for the publication in F1000 research. However, some parts of this data note needs information to explain the data set in a more detailed way before indexing.*

***My minor concerns are the following:***

***First comment**:*

- *Introduction section should be elaborated by explaining the benefits of a data set for researchers who study child marriages. The definitions of early marriage and the choice of using early marriage instead of child marriage should be explained. Since, in many data sets it is measured by the age of marriage before 18 or 15.*
- *The reference time should be included about the prevalence of child marriage of Ethiopia.*

**Authors’ Response:** **We greatly appreciate the reviewer’s helpful comment and suggestion**

 This dataset is used as a great source for researchers who study early marriage and interested in making use of it as a secondary use of their routinely collected data **.** It also enables to generalize the findings for a large community as it is huge in number and shows a trend of early marriage for more than a decade.

 Early marriage, or child marriage, is defined as the marriage or union between two people in which one or both parties are younger than 18 years of age (OHCHR, 2019). Marriages that take place before age 15 are considered “very early marriages.” (UNFPA, 2020). “Early marriage” has been interpreted, as synonymous with “child marriage” or as more inclusive as child marriage. 'Early' does not have to refer solely to age, however, and could be read to include other factors that would make a person unready to consent to marriage (sri, 2012). Although early is not explicitly defined to mean less than 18 years old, it is frequently found in that context. Several organizations like World Health Organization, UN for instance, use the term ‘early marriage’ consistently(UNICEF, 2005).

 The highest rate of child marriage is in sub-Saharan Africa, with 37 percent of young women marrying before age 18. According to the Ethiopian demography and health survey (EDHS) 2016, the national prevalence of early marriage was 58% (CSA, 2016).

***Second Comment***

*In the material and method section (it must be named as), more explanation is needed about the system of KERSA HDHSS. The aim of the system in general, sample size, how data is collected, what are the other specific demographic events, other variables and what are the limitations of the data set some of the questions that should be mentioned.*

**Authors’ Response** **: **Thank you, we appreciate the valuable suggestions. It is explained as suggested.

**Methods and Materials**

 Kersa HDSS is a demographic and health surveillance and research center established in 2007 by Haramaya University to serve as research center and source health and demographics data for Eastern Ethiopia, thus to create a framework for research at the community level and to be a platform for various health related research by the college of Health and Medical Sciences in Haramaya University. Kersa HDSS does monitoring demographic events such as birth, death, marital status change and migration; and health related conditions such as pregnancy, immunization, and morbidity among children and adults. The baseline census was conducted in 2007 and since then has been updated every 6 months, with registration of demographic and health events (Assefa *et al*., 2016). Data collectors who know the language of the community are permanently recruited and execute the data collection regularly. 

 This study used data from an open dynamic cohort that gives a leverage of huge set of data from exiting Health and Demographic Surveillance System (Kersa HDSS) with a sample size of (24,452) that helps to generalize the findings to eastern part of the country.

 KHDSS data collection tool lack some important exposure variables that help to assess the socio cultural factors; like social norms, reason for marriage, parental education, occupation and socioeconomic status of the parents, which had a significant effect on early marriage. Hence, could be considered as a limitation of this study.

**References**

 Assefa, N., Oljira, L., Baraki, N., Demena, M., Zelalem, D., Ashenafi, W., & Dedefo, M. (2016). HDSS profile: the Kersa health and demographic surveillance system. *International Journal of Epidemiology, 45*(1), 94-101.

 CSA. (2016). Ethiopia demographic and health survey. Central Statistical Agency Addis Ababa. *Ethiopia ICF International Calverton, Maryland, USA*.

 OHCHR. (2019). Child and forced marriage, including in humanitarian settings.

 sri. (2012). United Nations General Assembly Resolution 66/140, The Girl Child (2012), A/RES/66/140, p. 4; Beijing Platform for Action (1995), L. The Girl Child para. 259.

 UNFPA. (2020). Child marriage - Frequently Asked Questions.

 UNICEF. (2005). EARLY MARRIAGE: A HARMFUL TRADITIONAL PRACTICE: A STATISTICAL EXPLORATION (2005), p. 4. .

# Response

**Published:** 2022-01-07

**Authors:** Dureti Abdurahman

**Affiliations:** Haramaya University, Ethiopia

**Dear reviewer**

 We greatly appreciate the time and effort put forth to improve our paper. We benefited a lot from their insightful comments and suggestions. Based on the suggestions, we have provided a response to each comment as follows, and we will incorporate it on the main document.

***General comment***

*This data note describes the data set of Kersa Health and Surveillance System (Kersa HDSS) for the use of secondary analysis. It is appropriate for the publication in F1000 research. However, some parts of this data note needs information to explain the data set in a more detailed way before indexing.*

***My minor concerns are the following:***

***First comment**:*

- *Introduction section should be elaborated by explaining the benefits of a data set for researchers who study child marriages. The definitions of early marriage and the choice of using early marriage instead of child marriage should be explained. Since, in many data sets it is measured by the age of marriage before 18 or 15.|*
- *The reference time should be included about the prevalence of child marriage of Ethiopia.*

**Authors’ Response: **We greatly appreciate the reviewer’s helpful comment and suggestion.

 This dataset is used as a great source for researchers who study early marriage and interested in making use of it as a secondary use of their routinely collected data **.** It also enables to generalize the findings for a large community as it is huge in number and shows a trend of early marriage for more than a decade.

 Early marriage, or child marriage, is defined as the marriage or union between two people in which one or both parties are younger than 18 years of age (OHCHR, 2019). Marriages that take place before age 15 are considered “very early marriages.” (UNFPA, 2020). “Early marriage” has been interpreted, as synonymous with “child marriage” or as more inclusive as child marriage. 'Early' does not have to refer solely to age, however, and could be read to include other factors that would make a person unready to consent to marriage (sri, 2012). Although early is not explicitly defined to mean less than 18 years old, it is frequently found in that context. Several organizations like World Health Organization, UN for instance, use the term ‘early marriage’ consistently (UNICEF, 2005).

 The highest rate of child marriage is in sub-Saharan Africa, with 37 percent of young women marrying before age 18. According to the Ethiopian demography and health survey (EDHS) 2016, the national prevalence of early marriage was 58% (CSA, 2016).

***Second Comment***

*In the material and method section (it must be named as), more explanation is needed about the system of KERSA HDHSS. The aim of the system in general, sample size, how data is collected, what are the other specific demographic events, other variables and what are the limitations of the data set some of the questions that should be mentioned.*

**Authors’ Response** **: **Thank you, we appreciate the valuable suggestions. It is explained as suggested.

**Methods and Materials**

 Kersa HDSS is a demographic and health surveillance and research center established in 2007 by Haramaya University to serve as research center and source health and demographics data for Eastern Ethiopia, thus to create a framework for research at the community level and to be a platform for various health related research by the college of Health and Medical Sciences in Haramaya University. Kersa HDSS does monitoring demographic events such as birth, death, marital status change and migration; and health related conditions such as pregnancy, immunization, and morbidity among children and adults. The baseline census was conducted in 2007 and since then has been updated every 6 months, with registration of demographic and health events (Assefa *et al*., 2016). Data collectors who know the language of the community are permanently recruited and execute the data collection regularly. 

 This study used data from an open dynamic cohort that gives a leverage of huge set of data from exiting Health and Demographic Surveillance System (Kersa HDSS) with a sample size of (24,452) that helps to generalize the findings to eastern part of the country.

 KHDSS data collection tool lack some important exposure variables that help to assess the socio cultural factors; like social norms, reason for marriage, parental education, occupation and socioeconomic status of the parents, which had a significant effect on early marriage. Hence, could be considered as a limitation of this study.

**References**

 Assefa, N., Oljira, L., Baraki, N., Demena, M., Zelalem, D., Ashenafi, W., & Dedefo, M. (2016). HDSS profile: the Kersa health and demographic surveillance system. *International Journal of Epidemiology, 45*(1), 94-101.

 CSA. (2016). Ethiopia demographic and health survey. Central Statistical Agency Addis Ababa. *Ethiopia ICF International Calverton, Maryland, USA*.

 OHCHR. (2019). Child and forced marriage, including in humanitarian settings.

 sri. (2012). United Nations General Assembly Resolution 66/140, The Girl Child (2012), A/RES/66/140, p. 4; Beijing Platform for Action (1995), L. The Girl Child para. 259.

 UNFPA. (2020). Child marriage - Frequently Asked Questions.

 UNICEF. (2005). EARLY MARRIAGE: A HARMFUL TRADITIONAL PRACTICE: A STATISTICAL EXPLORATION (2005), p. 4. .

VIEW LESS
